# Introduction to *A Compendium of Strategies to Prevent Healthcare-Associated Infections In Acute-Care Hospitals: 2022 Updates*

**DOI:** 10.1017/ice.2023.158

**Published:** 2023-10

**Authors:** Deborah S. Yokoe, Sonali D. Advani, Deverick J. Anderson, Hilary M. Babcock, Michael Bell, Sean M. Berenholtz, Kristina A. Bryant, Niccolò Buetti, Michael S. Calderwood, David P. Calfee, Valerie M. Deloney, Erik R. Dubberke, Katherine D. Ellingson, Neil O. Fishman, Dale N. Gerding, Janet Glowicz, Mary K. Hayden, Keith S. Kaye, Larry K. Kociolek, Emily Landon, Elaine L. Larson, Anurag N. Malani, Jonas Marschall, Jennifer Meddings, Leonard A. Mermel, Payal K. Patel, Trish M. Perl, Kyle J. Popovich, Joshua K. Schaffzin, Edward Septimus, Kavita K. Trivedi, Robert A. Weinstein, Lisa L. Maragakis

**Affiliations:** 1 School of Medicine, UCSF Health-UCSF Medical Center, University of California, San Francisco, California, United States; 2 Duke University School of Medicine, Durham, North Carolina, United States; 3 BJC Healthcare, Washington University School of Medicine, St. Louis, Missouri, United States; 4 Centers for Disease Control and Prevention, Atlanta, Georgia, United States; 5 Johns Hopkins University, Baltimore, Maryland, United States; 6 University of Louisville School of Medicine, Norton Healthcare Louisville, Kentucky, United States; 7 Infection Control Programme, Geneva University Hospitals and Faculty of Medicine, World Health Organization Collaborating Center, Geneva, Switzerland; 8 IAME-U1137, Université Paris-Cité, INSERM, Paris, France; 9 Dartmouth Hitchcock Medical Center, Lebanon, New Hampshire, United States; 10 Weill Cornell Medicine, New York, New York, United States; 11 Society for Healthcare Epidemiology of America, Arlington, Virginia, United States; 12 Washington University School of Medicine, St. Louis, Missouri, United States; 13 College of Public Health, The University of Arizona, Tucson, Arizona, United States; 14 Hospital of the University of Pennsylvania, Penn Medicine, Philadelphia, Pennsylvania, United States; 15 Edward Hines Jr. Veterans’ Affairs Hospital, Hines, Illinois, United States; 16 Rush University Medical Center, Chicago, Illinois, United States; 17 Rutgers Robert Wood Johnson Medical School, New Brunswick, New Jersey, United States; 18 Northwestern University Feinberg School of Medicine, Chicago, Illinois, United States; 19 Ann & Robert H. Lurie Children’s Hospital of Chicago, Chicago, Illinois, United States; 20 The University of Chicago Medical Center, MacLean Center for Clinical Medical Ethics, Chicago, Illinois, United States; 21 Columbia School of Nursing, New York, New York, United States; 22 Trinity Health Michigan, Ann Arbor, Michigan, United States; 23 Bern University Hospital, University of Bern, Bern, Switzerland; 24 University of Michigan Medical School, Veterans’ Affairs Ann Arbor Healthcare System, Ann Arbor, Michigan, United States; 25 Warren Alpert Medical School of Brown University, Lifespan Hospital System, Providence, Rhode Island, United States; 26 Intermountain Healthcare, Salt Lake City, Utah, United States; 27 University of Texas Southwestern Medical Center, Dallas, Texas, United States; 28 Children’s Hospital of Eastern Ontario, University of Ottawa, Ottawa, Ontario, Canada; 29 Texas A&M College of Medicine, Houston, Texas, United States; 30 Harvard Pilgrim Health Care, Boston, Massachusetts, United States; 31 Alameda County Public Health Department, San Leandro, California, United States; 32 Cook County Health, Chicago, Illinois, United States; 33 Johns Hopkins University School of Medicine, The Johns Hopkins Hospital, Baltimore, Maryland, United States

## Abstract

Since the initial publication of *A Compendium of Strategies to Prevent Healthcare-Associated Infections in Acute Care Hospitals* in 2008, the prevention of healthcare-associated infections (HAIs) has continued to be a national priority. Progress in healthcare epidemiology, infection prevention, antimicrobial stewardship, and implementation science research has led to improvements in our understanding of effective strategies for HAI prevention. Despite these advances, HAIs continue to affect ∼1 of every 31 hospitalized patients,^1^ leading to substantial morbidity, mortality, and excess healthcare expenditures,^1^ and persistent gaps remain between what is recommended and what is practiced.

The widespread impact of the coronavirus disease 2019 (COVID-19) pandemic on HAI outcomes^2^ in acute-care hospitals has further highlighted the essential role of infection prevention programs and the critical importance of prioritizing efforts that can be sustained even in the face of resource requirements from COVID-19 and future infectious diseases crises.^3^

The *Compendium: 2022 Updates* document provides acute-care hospitals with up-to-date, practical expert guidance to assist in prioritizing and implementing HAI prevention efforts. It is the product of a highly collaborative effort led by the Society for Healthcare Epidemiology of America (SHEA), the Infectious Disease Society of America (IDSA), the Association for Professionals in Infection Control and Epidemiology (APIC), the American Hospital Association (AHA), and The Joint Commission, with major contributions from representatives of organizations and societies with content expertise, including the Centers for Disease Control and Prevention (CDC), the Pediatric Infectious Disease Society (PIDS), the Society for Critical Care Medicine (SCCM), the Society for Hospital Medicine (SHM), the Surgical Infection Society (SIS), and others.

Since the initial and updated publications of *A Compendium of Strategies to Prevent Healthcare-Associated Infections in Acute Care Hospitals* in 2008 and 2014,^4–^^10^ substantial progress in HAI prevention has been achieved through the combined efforts of federal, state, and local public health entities and healthcare facilities. These efforts have been supported by the US Department of Health and Human Services (HHS) National Action Plan to Prevent Health Care-Associated Infections (HAIs),^11^ in coordination with the Centers for Medicare and Medicaid Services (CMS) HAI reporting requirements and reimbursement penalties tied to healthcare facilities’ HAI performance regarding central-line–associated bloodstream infections (CLABSIs), catheter-associated urinary tract infections (CAUTIs), surgical-site infections (SSIs), methicillin-resistant *Staphylococcus aureus* (MRSA) bloodstream infections, and *Clostridioides difficile* infections (CDIs). Progress in healthcare epidemiology and implementation science research has led to improvements in our understanding of effective strategies for HAI prevention. Despite these advances, the Centers for Disease Control and Prevention (CDC) estimates that each day, ∼1 in 31 patients in US healthcare facilities^1^ contracts at least 1 infection in association with hospital care, leading to substantial morbidity, mortality, and excess healthcare expenditures.^1^

Based on HAI surveillance data collected by the CDC National Healthcare Safety Network (NHSN), substantial improvements were achieved in preventing CLABSI, CAUTI, CDI, and SSI between 2015 and 2019, including national decreases of 31% for CLABSI, 26% for CAUTI, 42% for CDI, and 7% for SSI.^11^

That positive trend was reversed starting in 2020.^12^ The coronavirus disease 2019 (COVID-19) pandemic created unprecedented challenges, affecting the ability of healthcare facilities to consistently maintain practices essential for HAI prevention and resulting in negative impacts on HAI outcomes as hospitals responded to surges of patients with COVID-19. The pandemic strained available healthcare resources including hospital beds, staffing, and medical supplies and diverted HAI prevention resources toward COVID-19 response efforts. The effect on HAI risk is reflected in the results of several studies^13–^^17^ as well as the CDC’s 2020 and 2021 National and State Healthcare-Associated Infections Progress Reports,^3,16,^^17^ which demonstrated substantial increases in CLABSI rates (33% increase overall and 65% increase in ICUs), CAUTI rates (20% increase in ICUs), VAE rates (51% increase overall), and MRSA bacteremia rates (31% increase) in 2021 compared to 2019. These changes highlight the importance of tools such as the *Compendium*, which can be used by acute-care facilities to prioritize and implement HAI prevention strategies that can lead to improvements in outcomes and that are sustainable in public health crises.

## Compendium: 2022 Updates

Since its initial publication in 2008, the *Compendium* has provided acute-care hospitals with current, practical, and concise expert guidance to assist in prioritizing and implementing HAI prevention strategies.

Consistent with the 2008 and 2014 publications, the recommendations included in the *Compendium: 2022 Updates* are based on previously published HAI prevention guidelines available from organizations, including the Healthcare Infection Control Practices Advisory Committee (HICPAC), the CDC, Society for Healthcare Epidemiology of America (SHEA), the Infectious Disease Society of America (IDSA), the Association for Professionals in Infection Control and Epidemiology (APIC), relevant published literature, consensus of the author panels’ members, and multiorganizational review and approval of recommendations.

The *Compendium: 2022 Updates* authors utilized the systematic literature review process described in the *SHEA Handbook for SHEA-Sponsored Guidelines and Expert Guidance Documents*.^18^ The *Compendium* is not meant to supplant previously published guidelines and systematic reviews. The *Compendium: 2022 Updates* includes 8 articles: 6 focused on prevention of specific types of HAIs, 1 section focused on hand hygiene improvement strategies, and a new section focused on implementation strategies relevant to HAI prevention. Except for the implementation document, each section contains a statement of concern, a brief summary of surveillance and prevention approaches, recommended infection prevention interventions, proposed performance measures, and examples of implementation strategies for consideration.

Each infection prevention recommendation is given a level of evidence rating (low, moderate, or high level of evidence) adapted from criteria utilized by the Grades of Recommendation, Assessment, Development, and Evaluation (GRADE) system,^19^ the Canadian Task Force on Preventive Health Care,^20^ and the HICPAC Evidence and Guideline Categorization Scheme (Table 1).^21^ The Implementation article provides a statement of concern, followed by information about approaches to measurement, conceptual models and frameworks, and future needs in development, adaptation, and utilization of implementation models and frameworks for infection prevention and control.

**Table 1.** Quality of Evidence

HIGH	Highly confident that the true effect lies close to that of the estimated size and direction of the effect. Evidence is rated as “high” quality when there are a wide range of studies with no major limitations, there is little variation between studies, and the summary estimate has a narrow confidence interval.
MODERATE	The true effect is likely to be close to the estimated size and direction of the effect, but there is a possibility that it is substantially different. Evidence is rated as “moderate” quality when there are only a few studies and some have limitations but not major flaws, there is some variation between studies, or the confidence interval of the summary estimate is wide.
LOW	The true effect may be substantially different from the estimated size and direction of the effect. Evidence is rated as “low” quality when supporting studies have major flaws, there is important variation between studies, the confidence interval of the summary estimate is very wide, or there are no rigorous studies.

Based on the CDC Healthcare Infection Control Practices Advisory Committee (HICPAC) “Update to the Centers for Disease Control and Prevention and the Healthcare Infection Control Practices Advisory Committee Recommendations Categorization Scheme for Infection Control and Prevention Guideline Recommendations” (October 2019),^21^ the Grades of Recommendation, Assessment, Development, and Evaluation (GRADE),^19^ and the Canadian Task Force on Preventive Health Care.^20^

Compendium recommendations are categorized as follows:

1. Essential practices (previously called “basic practices” and renamed to highlight their foundational importance for HAI prevention) that should be adopted by all acute-care hospitals unless a clear and compelling rationale for an alternative approach is present.
2. Additional approaches (previously called “special approaches”) that can be considered for use in locations and/or hospital patient populations when HAIs are not controlled after implementation of essential practices.

The decision to categorize a recommendation as an essential practice versus an additional approach was made through consensus of the author panel with input from the Expert Panel.

In general, essential practices are supported by high to moderate-quality evidence, but some recommendations with low or moderate-quality evidence are classified as essential practices when high-quality evidence was determined to be impossible to obtain and anticipated benefits strongly outweighed potential harms based on the assessment of the author panel and Expert Panel (or, in the case of a negative recommendation, that harms clearly exceeded benefits). Recommendations classified as additional approaches may be supported by low, moderate, or high-quality evidence, with the author panel and Expert Panel assessing that the benefits of the recommended approach are likely to exceed the harms (or, in the case of a negative recommendation, that harms are likely to exceed benefits). The following criteria inform the evidence level identified for a recommendation classified as an additional approach:

1. There was high-quality evidence, but the benefit–harm balance was not clearly tipped in one direction.
2. The evidence was weak enough to cast doubt on whether the recommendation would consistently lead to benefit.
3. The likelihood of benefit for a specific patient population or clinical situation was extrapolated from relatively high-quality evidence demonstrating impact on other patient populations or in other clinical situations (eg, evidence obtained during outbreaks used to support probable benefit during endemic periods).
4. The impact of the specific intervention was difficult to disentangle from the impact of other simultaneously implemented interventions (eg, studies evaluating “bundled” practices).
5. There appeared to be benefit based on available evidence, but the benefit–harm balance may change with further research.

Hospitals can prioritize their efforts by initially focusing on implementing essential practices. If HAI surveillance or a risk assessment suggests that targets are not being met by using the essential practices, hospitals should then consider adopting some or all of the additional approaches. These can be implemented in specific locations or patient populations, or can be implemented hospital-wide, depending on outcome data, risk assessment, and/or local requirements.

## Methods

SHEA convened 8 author panels to develop the *Compendium: 2022 Updates*; the overall coordination of the *Compendium: 2022 Updates* was directed by leads appointed by SHEA and IDSA (D. Yokoe and L. Maragakis) and by the SHEA Guidelines staff lead (V. Deloney). The SHEA Guidelines Committee and Board of Trustees and the IDSA Standards and Practice Guidelines Committee recruited 2–3 subject-matter experts in the prevention of 6 HAIs and the prevention strategy of hand hygiene to lead 12 to 14-member author panels for each *Compendium* section. SHEA and IDSA appointed 2–3 individuals from each panel to author the Implementation section, a new addition in the *Compendium: 2022 Updates*.

In addition to SHEA, IDSA, APIC, and CDC, panels included representation from The Joint Commission, The Association of periOperative Registered Nurses (AORN), Society of Infectious Diseases Pharmacists (SIDP), the Surgical Infection Society (SIS), the Pediatric Infectious Diseases Society (PIDS), and others.

## Literature review and analysis

SHEA hired a consultant medical librarian (J. Waters), who developed a comprehensive search strategy for PubMed and Embase (January 2012–July 2019, updated to August 2021). Articles’ abstracts were reviewed by panel members. Each abstract was reviewed by at least 2 reviewers using the abstract management software Covidence (Melbourne, Australia), and selected abstracts were reviewed as full text. In July 2021, the Compendium Lead Authors group voted to update the literature findings, and the librarian reran the search to update it to August 2021. Panel members screened the articles yielded by the search via Covidence and incorporated relevant references (see Executive Summary Supplementary Materials online).

Author Panel members for each *Compendium* section met as needed via video conference to develop and discuss recommendations; to rank of the quality of evidence for these recommendations; and to classify the recommendations as essential practices, additional approaches, practices that should not be a routine part of prevention, or unresolved issues. Panel members reviewed and approved each document and its recommendations.

## Review and approval process

An Advisory Group consisting of representatives from the 5 major partnering organizations (SHEA, IDSA, APIC, The Joint Commission, and AHA) provided broad oversight over the process (Table 3). Participants complied with the SHEA and IDSA policies on conflict-of-interest disclosure.

**Table 2.** Level of Recommendation

Level of Recommendation	Evidence Level	Implied Obligation
**Essential practice**: Panel members are confident the benefits of the recommended approach clearly exceed the harms (or, in the case of a negative recommendation, that the harms clearly exceed the benefits).	In general, high or moderate-quality evidence (Table 1) or lesser evidence or expert opinion when high-quality evidence is impossible to obtain and the anticipated benefits strongly outweigh the harms.	In general, healthcare personnel and facilities “should” implement the recommended approach unless a clear and compelling rationale for an alternative approach is present.
**Additional approach**: Panel members have determined that the benefits of the recommended approach are likely to exceed the harms (or, in the case of a negative recommendation, that the harms are likely to exceed the benefits).	• In general, may be supported by either low-, moderate-, or high-quality evidence. • There is high-quality evidence, but the benefit–harm balance is not clearly tipped in one direction. • The evidence is weak enough to cast doubt on whether the recommendation will consistently lead to benefit. • The likelihood of benefit for a specific patient population or clinical situation is extrapolated from relatively high-quality evidence demonstrating impact on other patient populations or in other clinical situations (eg, evidence obtained during outbreaks used to support probable benefit during endemic periods). • The impact of the specific intervention is difficult to disentangle from the impact of other simultaneously implemented interventions (eg, studies evaluating “bundled” practices). • There appears to be benefit based on available evidence, but the benefit/harm balance may change with further research. • Benefit is most likely if the intervention is used as a supplemental measure in addition to essential practices.	Healthcare personnel and facilities “could,” or “may consider” implementing the recommended approach. The degree of appropriateness may vary depending on the benefit–harm balance for the specific setting.
**Unresolved issue**: Panel members agree that there is both a lack of pertinent evidence and unclear balance between benefits and harms.		

**Table 3.** Compendium Leadership

Society for Healthcare Epidemiology of America (SHEA) co-chair	Deborah S. Yokoe MD, MPH
Infectious Disease Society of America (IDSA) co-chair	Lisa L. Maragakis MD, MPH
Compendium Advisory Group	Valerie M. Deloney MBA (SHEA) Kristy Weinshel MBA (SHEA) Emily Sickbert-Bennett PhD, MS, CIC (SHEA Guidelines Committee Chair) Genet Demisashi (IDSA) Shanina Knighton PhD, RN, CIC (APIC, 2022–2023) Sylvia Quevedo MS (APIC, 2018–2022) Nancy Foster (AHA, 2018–2022) Kavita Bhat MD, MPH, CPHQ (AHA, 2023) Margaret VanAmringe MHS (The Joint Commission) David Classen MD, MS (2008 IDSA *Compendium* co-chair)
Compendium Expert Panel	Hilary M. Babcock MD, MPH Michael Bell MD Kristina A. Bryant MD Neil O. Fishman MD Mary K. Hayden MD Elaine L. Larson PhD, RN, CIC Anurag Malani MD Trish M. Perl MD, MSc Edward Septimus MD Robert A. Weinstein MD
Compendium Lead Authors	Payal K. Patel MD, MPH (CAUTI) Jennifer Meddings MD, MSc (CAUTI) Sonali D. Advani MBBS, MPH (CAUTI) Niccolò Buetti MD, MSc, PhD (CLABSI) Jonas Marschall MD, MSc (CLABSI) Leonard A. Mermel DO, ScM (CLABSI) Larry K. Kociolek MD, MSCI (*C. difficile* Infection) Erik R. Dubberke MD, MSPH (*C. difficile* Infection) Dale N. Gerding MD (*C. difficile* Infection) Janet Glowicz PhD, RN, CIC (Hand Hygiene) Katherine D. Ellingson PhD (Hand Hygiene) Emily Landon MD (Hand Hygiene) Kyle J. Popovich MD, MS (MRSA) David P. Calfee MD, MS (MRSA) Michael S. Calderwood MD, MPH (SSI) Deverick J. Anderson MD, MPH (SSI) Keith S. Kaye MD, MPH (SSI) Michael Klompas MD, MPH (VAP, VAE, NV-HAP) Sean M. Berenholtz MD, MHS (VAP, VAE, NV-HAP) Kavita K. Trivedi MD (Implementation) Joshua K. Schaffzin MD, PhD (Implementation) Sean M. Berenholtz MD, MHS (Implementation)

The Expert Panel consisting of members with broad healthcare epidemiology and infection prevention expertise reviewed the draft manuscripts after the author panels reached consensus on the recommendations. These panel members provided input regarding recommendations and their levels of evidence. The Author Panels revised the draft recommendations to incorporate the Expert Panel’s input. Subsequently, the Expert Panel, the *Compendium* Partners, collaborating professional organizations, and the CDC reviewed and approved the documents.

Finally, the SHEA Guidelines Committee, the IDSA Standards and Practice Guidelines Committee, the Boards of SHEA, IDSA, and APIC, and AHA and The Joint Commission reviewed and approved the documents. In addition to the 5 *Compendium* partners, endorsing and supporting organizations are acknowledged in Table 4.

**Table 4.** *Compendium: 2022 Updates* Endorsing and Supporting Organizations

**Endorsing** **Organizations** Provided formal review and approval of the articles that compose the *Compendium: 2022 Updates*	The Society for Healthcare Epidemiology of America (SHEA) The Infectious Diseases Society of America (IDSA) The Association for Professionals in Infection Control and Epidemiology (APIC) The American Hospital Association (AHA) The Joint Commission American Society of Health System Pharmacists (ASHP) for CLABSI, VAP/VAE/NV-HAP, Hand Hygiene, *C. difficile* Infection, SSI, and Implementation Anesthesia Patient Safety Foundation (APSF) National Foundation for Infectious Diseases (NFID) for MRSA, CAUTI, SSI, and *C. difficile* Infection Pediatric Infectious Diseases Society (PIDS) Society of Infectious Diseases Pharmacists (SIDP) for VAP/VAE/NV-HAP and Implementation Surgical Infection Society (SIS)
**Supporting Organizations** *Provided general, nonfinancial support for the Compendium: 2022 Updates*	The Council of State and Territorial Epidemiologists (CSTE) The National Association of County and City Health Officials (NACCHO) Society of Critical Care Medicine (SCCM) Society of Infectious Diseases Pharmacists (SIDP)
Organizations that endorsed or provided support for the *Compendium: 2022 Updates* after the publication of this document are listed on the SHEA website: www.shea-online.org.	

## Disclosure of conflicts of interest

All members of the *Compendium* Writing Panels, Expert Panel, and Advisory Group complied with SHEA policies on conflicts of interest, which require disclosure of any financial or other interest within the past 2 years that might be construed as constituting an actual, potential, or apparent conflict. SHEA requires full disclosure of all relationships, including employment, consultancies, stock ownership, honoraria, research funding, expert testimony, and membership on company advisory committees, regardless of relevancy to the topic. Disclosed relationships that are associated with potential conflicts of interest are evaluated in a review process that includes the SHEA Conflict of Interest Committee and may include the Board of Trustees and editors of *Infection Control and Hospital Epidemiology*. The assessment of disclosed relationships for possible conflicts of interest has been based on the relative weight of the financial relationship (ie, monetary amount) and the relevance of the relationship (ie, the degree to which an association might reasonably be interpreted by an independent observer as related to the topic or recommendation of consideration). Compendium participants with potential conflicts were required to submit a plan detailing the process that would be used to avoid any effects of these conflicts. Decisions were made on a case-by-case basis as to whether an individual’s role should be limited because of a conflict. Potential conflicts are listed in the Acknowledgments of the individual articles and in the Executive Summary.

## Mechanism for updating the *Compendium*

At ∼5-year intervals, the SHEA Guidelines Committee, with the *Compendium* Author Panel leads and other appropriate content experts will assess the need for updates to *Compendium* recommendations. Decisions regarding the timing of future *Compendium* updates will be made by SHEA in collaboration with IDSA, APIC, AHA, and The Joint Commission.
